# Addictive Internet Gaming Usage among Korean Adolescents before and after the Outbreak of the COVID-19 Pandemic: A Comparison of the Latent Profiles in 2018 and 2020

**DOI:** 10.3390/ijerph18147275

**Published:** 2021-07-07

**Authors:** Dongil Kim, Junwon Lee

**Affiliations:** Department of Education, College of Education, Seoul National University, Seoul 08826, Korea; dikimedu@snu.ac.kr

**Keywords:** internet gaming disorder, adolescents, COVID-19, gameplay time, Korea

## Abstract

Since the outbreak of the COVID-19 pandemic, the heightened risk of school closures and mental disorders has made adolescents particularly vulnerable to developing internet gaming disorder (IGD). There have been reports of increased time spent playing games on the internet among adolescents during the pandemic, and the risk of developing IGD may be higher for adolescents in South Korea as the majority of them play games on the internet. However, to the best of our knowledge, no studies have examined the impact of the pandemic on adolescents’ internet gaming behavior in South Korea. This study aimed to explore the different profiles of addictive internet gaming behavior among adolescents before and after the outbreak of the COVID-19 pandemic and examine how the pandemic influenced addictive internet gaming usage and time spent playing games on the internet. Nationally representative survey data from the Ministry of Gender Equality and Family with 3040 and 2906 responses from 2018 and 2020, respectively, were analyzed. Using seven factors of a maladaptive gaming usage scale (tolerance, withdrawal, excessive usage, control impairment, compulsive usage, neglecting daily activity, and gaming despite negative consequence), a four-profile model was selected in both 2018 and 2020 for latent profile analysis: ‘casual’ gamer, ‘moderate’ gamer, ‘potential-risk’ gamer and ‘addictive’ gamer. The results from the two-way ANCOVA showed significant interaction between the cohorts (2018 cohort vs. 2020 cohort) and the four profiles on addictive internet gaming usage (F = 119.747, *p* < 0.001, η^2^ = 0.05), including time spent playing internet games on a PC (F = 22.893, *p* < 0.001, η^2^ = 0.013), and time spent playing games on a mobile phone (F = 3.245, *p* < 0.05, η^2^ = 0.02). The results indicated that the increase of addictive internet gaming usage and gameplay time differed by profile. The results imply that the increase in gameplay time was higher for profiles with higher scores in addictive internet gaming usage for internet games played on a PC while the relationship was not obvious for games played on a mobile phone. Despite the statistical significance, there was only 1.2% to 4.9% of mean difference in addictive internet gaming usage between the 2018 and 2020 cohorts, which implies little clinical significance. While adolescents of the four profiles showed no significant signs of increased addictive internet gaming usage, the addictive gamer profile demonstrated a significant increase in game time after COVID-19.

## 1. Introduction

Since the outbreak of the COVID-19 pandemic in January 2020, our daily lives have been disrupted due to social distancing and quarantine, along with the fear of infection and stress, resulting in an increased prevalence of psychological disorders, such as depression, anxiety, suicidal behavior, and heightened stress [1,2,3,4]. Additionally, a recent study conducted in China reported anxiety, depression, and stress to be the three most prevalent psychological symptoms of adolescents during the COVID-19 school closure in China [5], and another study reported that one-third of the children in Italy, Spain, and Portugal experienced anxiety during the COVID-19 quarantine [6].

Along with the increased risk of having psychological symptoms of depression, anxiety, and stress due to the impact of the pandemic, scholars have warned about the increased risk of developing a behavioral addiction, such as internet gaming disorder (IGD), which is known to be more prevalent in males [7,8,9,10,11], in particular among adolescents [12,13]. The Diagnostic and Statistical Manual of Mental Disorders 5 (DSM-5) outlines the following symptoms as criteria for IGD: preoccupation, withdrawal, tolerance, failure to reduce or stop gaming, neglecting other activities, continuing gaming despite problems, deception, gaming to escape, and relationship risk due to excessive gaming [14]. To be diagnosed with IGD, a person should exhibit at least five of the symptoms for at least twelve months. Similarly, gaming disorder (GD), as classified by the 11th edition of the International Classification of Disease (ICD-11), comprises the following three symptoms: impaired control, increased priority given to gaming, and continued gaming despite negative consequences [15]; these symptoms focus on impairment of function while excluding biological aspects of addiction such as tolerance and withdrawal [16]. According to the ICD-11, a person should exhibit the aforementioned three symptoms for at least twelve months to be diagnosed with GD.

The relationship between psychological problems and IGD is reported globally [7,9], implying a heightened risk of adolescents developing IGD during the COVID-19 pandemic. One study conducted in Iran suggested that IGD significantly affected depression and anxiety among adolescents during the COVID-19 pandemic [17], and a recent study conducted in China reported an increased prevalence of IGD among adolescents after the outbreak of the COVID-19 pandemic [18]. In Italy, anxiety was found to be a predictor for online videogame and addiction [19], and a study from Japan indicated an increased prevalence of IGD, especially in the younger population [20]. In addition, one study from Hong Kong indicated that female children and adolescents felt more loneliness during the pandemic, while showing a significant relationship between loneliness and gaming addiction [21].

While internet gaming can be used as a means to relieve stressful situations during the COVID-19 [22] pandemic, most adolescents enjoy gaming in a healthy manner, resulting in positive outcomes such as a sense of achievement, friendship, and community [23,24,25,26]. During the COVID-19 pandemic, online gaming was found to relieve emotional distress due to social isolation [27], provide social support [28], and reduce loneliness [29]. However, the negative impacts of IGD are clear and should not be overlooked [30]. IGD can result in insomnia [17] and impairment in interpersonal relationships, education, and occupation [22]. Due to school closures, limited extracurricular activity, and social distancing during the COVID-19 pandemic, adolescents were forced to spend more time at home; this has since led to increased time spent partaking in internet gaming [31,32]. The environment of the pandemic induced an increase in internet usage, which may increase the risk of developing addictive behaviors in adolescents that persist throughout their lifespan [33]. One study from China reported that problematic gaming was a significant mediator of a relationship between psychological distress and time spent on internet related activities during the COVID-19 pandemic [34]. According to the interaction of the person-affect-cognition-execution (I-PACE) model [35], addiction develops from the interaction of the context (isolation and stress due to the pandemic), personal reaction (playing games on the internet to reduce stress and feel relieved), and consequences (neural changes, habituation, and compulsion) of repeated behaviors [35]. During the COVID-19 pandemic, playing games on the internet in moderation was viewed as a coping mechanism to reduce stress. However, there was a possibility of excessively playing games on the internet as isolation and social distancing severely limit available activities to reduce stress; this in turn accelerates the development of addictive behavior into a ‘later stage’ of addiction [35]. A longitudinal study on IGD in China used the I-PACE model to examine the relationship between emotional intelligence and depressive symptoms, coping flexibility, and IGD [36].

The impact of the pandemic on IGD may be greater for adolescents in South Korea due to a very high proportion of gamers—as high as 91.5%—among the adolescent population [37]. The results of the nationally representative survey conducted by the Korea Creative Agency (2020) indicated that more than half of the adolescents confirmed that the time spent playing games on the internet increased during the COVID-19 pandemic [37].

Although the time spent on internet gaming increased during the pandemic among South Korean adolescents, it is not certain whether IGD symptoms also increased compared with periods before the pandemic. Excessive internet usage has been known as an indicator of behavioral addiction since the conceptualization of internet addiction [38]. The deficient self-regulation perspective argues that excessive internet usage is a result of addictive internet usage [39]. Similarly, the I-PACE model explains that repeated gratification (such as relieving stress) of internet gaming behavior can change coping styles and reward expectancy; this may lead to an increased urge to play games on the internet when stressed and a weakened inhibitory response, which in turn, may increase the frequency of playing games and gameplay time [35]. Several studies suggest a strong relationship between time spent playing games on the internet and IGD [40,41,42]. However, some studies suggest a weak or no relationship, whereby the negative consequences of intensive gaming are not equal to those of pathological gaming [43,44,45,46]. One example of a highly involved, but not pathological, gamer is a professional gamer. An MRI study of the brain indicates that professional gamers have different volumes of gray matter compared to people with an online game addiction [47].

Moreover, the increasing trend in play time may influence adolescents differently. In a recent study, the IGD group exhibited higher gameplay time than the non-IGD group [42]. In a global study on the development of the IGD scale, five latent groups emerged with symptoms based on IGD in the DSM-5 [48]. Another IGD scale development study conducted in the Netherlands reported three latent groups based on the nine criteria of the DSM-5 [8]. In accordance with the I-PACE model, gameplay time in adolescents with more severe IGD symptoms might have increased further during the COVID-19 pandemic than in other healthier or non-severe groups of adolescents [35]. Therefore, the impact of the COVID-19 pandemic on adolescents’ addictive internet gaming behavior requires further investigation.

The aim of this study was to answer the following five research questions. First, do unique latent profiles of addictive internet gaming exist among South Korean adolescents; second, if unique latent profiles exist, do their memberships differ by gender; third, do total addictive internet game usage scores differ by profile membership and year; fourth, does internet gameplay time on a PC differ by profile membership and year; fifth, does gameplay time on mobile phones differ by profile membership and year.

## 2. Materials and Methods

### 2.1. Materials

The datasets analyzed in this study were obtained as secondary data from a nationally representative survey from the Ministry of Gender Equality and Family of South Korea, conducted biennially. The survey was approved by the Ministry of Gender Equality and Family and was conducted by the National Youth Policy Institute, a government- funded research institute. Data were collected using survey questionnaires distributed and collected by trained individuals from a registered research company from November to December in 2018 and 2020, approximately one year before and one year after the outbreak of the COVID-19 pandemic. Elementary students from grades four to six and middle school students from all seven provinces of South Korea were recruited for the survey. In total, 3317 and 3149 responses were collected in 2018 and 2020, respectively. After excluding survey responses with missing data using listwise deletion, 3040 responses from 2018, and 2906 responses from 2020 were analyzed [49,50]. In 2018, 48.3% of the respondents were female and 51.8% were elementary students with a mean age of 13.46 years (SD = 1.71); in 2020, 48.5% of the respondents were female and 47.1% were elementary students with a mean age of 13.62 years (SD = 1.71).

### 2.2. Measures

#### 2.2.1. Addictive Internet Gaming Usage

Addictive internet gaming usage was measured using the maladaptive game use scale (MGUS) [51]. The scale measures addictive gaming usage over the past year using a four-point Likert scale ranging from 1 (strongly disagree) to 4 (strongly agree). During the survey in 2018 and 2020, participants were instructed to answer the questionnaire specifically about internet gaming. The scale measures seven factors: tolerance (requiring longer playing time to feel the same amount of satisfaction), withdrawal (reducing online gameplaying time induces anxiety), excessive usage (playing online games longer than planned most of the time), control impairment (attempts to reduce online gaming ends up failing), compulsive usage (playing online games every day), neglecting daily activity (academic performance decreasing significantly) and gaming despite negative consequences (continue playing online games despite a conflict with parents), all of which include most of the criteria of IGD in DSM-V and all three criteria of GD in ICD-11. A score of nine or above in three or more factors indicated game addiction. Each factor was measured using three questions with a total of twenty-one questions. The total possible score ranged from twenty-one to eighty-four and the score of each factor ranged from three to twelve. The reported reliability of this scale was 0.92 [51]. In this study, the reliability was 0.95 for 2018 and 0.94 for 2020.

#### 2.2.2. Time Spent Playing Internet Games on a PC and Mobile Phones

The average gameplay time was measured as the average time spent playing internet games on a PC and games on a mobile phone during weekdays. The amount of play time was measured with two single open-ended questions: “On average, how much time do you spend playing Internet games on your PC on a weekday?” and “On average, how much time do you spend playing internet games on your mobile on a weekday?” Empty space was provided to record gameplay time in hours and minutes, which was then converted into total minutes. Since differences in characteristics have been reported between internet games played on a PC and games played on a mobile phone, play times of the two were evaluated separately. While internet games played on a PC, such as World of Warcraft, require a gamer to invest a large amount of time and concentration [25], games played on a mobile phone usually do not require prolonged involvement. Further, differences were identified between internet gaming using PCs and smartphones in a previous study in South Korea [52].

### 2.3. Data Analysis

Latent profile analysis (LPA) was used to estimate latent profiles of addictive internet gaming behavior of adolescents using seven factors of the MGUS in the 2018 and 2020 cohorts. To examine the effect of gender as a predictor of profile membership, a multinomial analysis was performed using the R3step procedure [53]. To compare the differences in time spent on gaming and addictive behaviors among profiles from the 2018 and 2020 cohorts, a two-way analysis of covariance (ANCOVA) was used after controlling for school level and gender. The post hoc test of the main effect was performed using the Bonferroni correction. LPA was performed using Mplus 8.2 software and ANCOVA was performed using SPSS 21.0 software.

## 3. Results

### 3.1. Correlation Analysis between MGUS Factors and Total Gaming Time

To explore the relationship among the seven MGUS factors and total gaming time in both 2018 and 2020, correlation analysis was conducted. The result indicted that all seven factors and total gaming time had significant positive correlation (*p* < 0.01). The correlation between total gaming time and the seven factors ranged from 0.225 to 0.358. The correlation between the seven factors ranged from 0.493 to 0.721. The results are presented in Table 1.

### 3.2. Latent Profile Analysis

To determine the optimal number of latent profiles, Akaike’s information criterion (AIC), Bayesian information criterion (BIC), sample size adjusted BIC (SABIC), entropy, Lo-Mendell-Rubin adjusted likelihood ratio test (MLR-LRT), bootstrap likelihood ratio test (BLRT), parsimony, and interpretability were considered. From previous studies, the number of profiles expected ranged from three to five [8,48]. AIC, BIC, and SABIC provided relative fit indexes and a smaller value indicated a better fit of the model [54]. Entropy examined classification accuracy with values ranging from zero to one, with higher scores representing higher accuracy [55]. The likelihood ratio test compared models by assessing whether increasing a class by one significantly improved the fit of the models [55]. Six profiles were estimated for the 2018 and 2020 cohorts. The fit indexes and results of the likelihood ratio test are presented in Table 2.

Considering the statistical criteria, models with four and five profiles emerged as models with the optimal number of profiles. AIC, BIC, and SABIC improved as the number of profiles increased and the BLRT also indicated that as the number of profiles increased the statistical fit improved significantly. For the LMR-LRT results, the model fit improved significantly until the four-profile model of the 2018 cohort and nearly significant improvement was observed in the four-profile model of the 2020 cohort. All models showed an entropy higher than 0.8, indicating adequate classification accuracy. In the five-profile model, one of the classes from the four-profile model diverged into two different classes which caused a challenging interpretation. Taking interpretability and parsimony into account, a four-profile model was selected over a five-profile model. Compared to the three-profile model, the four-profile model showed better fit in all three fit indexes, and the LMR-LRT and BLRT also favored the four-profile model. In addition, the membership of the three-profile model was 63.8%, 31.2%, and 5% in the 2018 cohort and 58.8%, 35.6% and 5.6% in the 2020 cohort, while class two in the three-profile model was divided into class two and three in the four-profile model, providing more information. The interpretability of the six-profile models in the 2018 and 2020 cohorts were low due to the complexity. Thus, considering the model fit, interpretability, and parsimony, the four-profile model was selected as the optimal model for both the 2018 and 2020 cohorts.

In the 2018 cohort, Profile 1 included 53.7%, Profile 2 included 20.8%, Profile 3 included 21.9%, and Profile 4 included 3.5% of the sample. In the 2020 cohort, Profile 1 included 50%, Profile 2 included 21.5%, Profile 3 included 26.2%, and Profile 4 included 2.3% of the sample. The distribution of addictive internet gaming behavior of each profile in the 2018 and 2020 cohorts were highly similar; thus, the same profile names were used. Profile 1 exhibited almost no addictive behaviors in all seven factors and the total internet gameplay time was significantly lower than the average play time of each year’s cohort; thus, this profile was named the ‘casual’ gamer profile. Profile 2 was named the ‘potential-risk’ gamer profile as addictive behaviors were mildly present with scores clustered around six, and the total internet gameplay time was similar to the average playing time of each year’s cohort. Profile 3 was named the ‘moderate’ gamer profile with overall low scores for all seven factors, and the total internet gameplay time was slightly lower than the average play time of each year’s cohort. Profile 4 was named the ‘addictive’ gamer profile with five to four factors with scores over nine, which is classified as addiction according to the MGUS. This profile met the diagnosis criteria of IGD and ICD. In addition, the total internet gameplay time of the ‘addictive’ gamer profile was significantly higher than the average play time of each year’s cohort.

The characteristics and proportion of profiles of the present study are similar to the results of previous studies conducted in the Netherlands and other countries [8,48]. In a Dutch study [6], three profiles were chosen, and the normal gamer profile was similar to the ‘casual’ and ‘moderate’ gamer profiles, the risky gamer profile was similar to the ‘potential-risk’ gamer profile, and the disordered profile was similar to the ‘addictive’ gamer profile. In the study conducted by Pontes et al. [48], other profiles were similar to those in this study, while the ‘potential-risk’ profile was divided into the low-risk, high-engagement gamer and at-risk, high-engagement gamer profiles.

**Figure 1:**
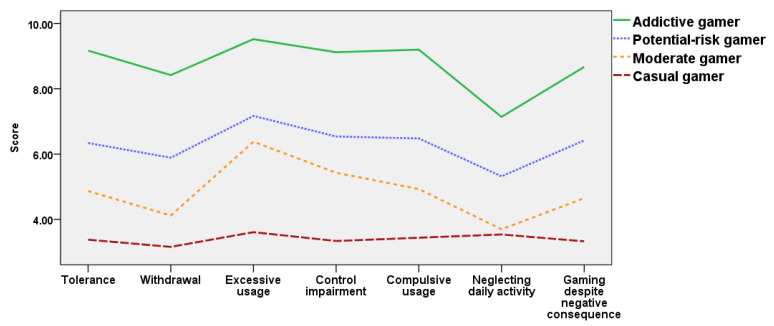
Profiles of addictive internet gaming usage of the 2018 cohort.

**Figure 2:**
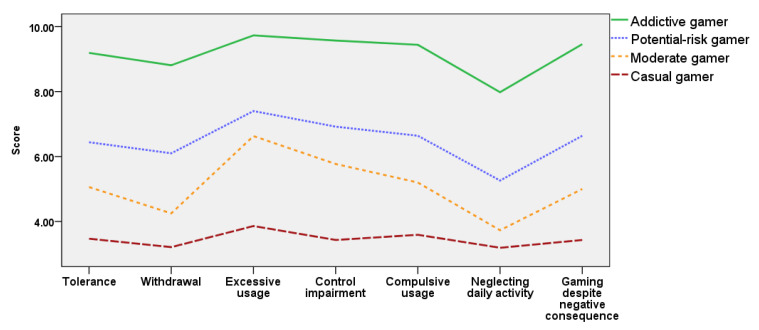
Profiles of addictive internet gaming usage of the 2020 cohort.

Although names were given to distinguish the four emergent profiles from each other, naming does not convey meaning other than indicating differences between addictive internet game usage and internet gameplay time. The addictive internet gaming usage scores of profiles and standard errors are presented in Table 3 and illustrated in Figure 1 and Figure 2.

### 3.3. Gender as a Predictor of Profile Membership

The association between gender and profile membership of the 2018 and 2020 cohorts was examined using a multinomial analysis. The results of the 2018 cohort showed that male respondents were more likely to be classified as ‘moderate’ gamers (OR = 2.00, *p* = 0.000), ‘potential-risk’ gamers (OR = 3.19, *p* = 0.000), or ‘addictive’ gamers (OR = 3.01, *p* = 0.015). The results of the 2020 cohort also showed that male respondents were more likely to be classified as ‘moderate’ gamers (OR = 1.74, *p* = 0.000), ‘potential-risk’ gamers (OR = 2.51, *p* = 0.000), or ‘addictive’ gamers (OR = 6.19, *p* = 0.002). Overall, male respondents had a higher chance of being classified into a profile with a higher addictive internet gaming usage score, and male respondents of the 2020 cohort were at greater risk of being classified as ‘addictive’ gamers.

### 3.4. Addictive Internet Gaming Usage, PC Internet Gaming Time and Mobile Gaming Time

**Figure 3:**
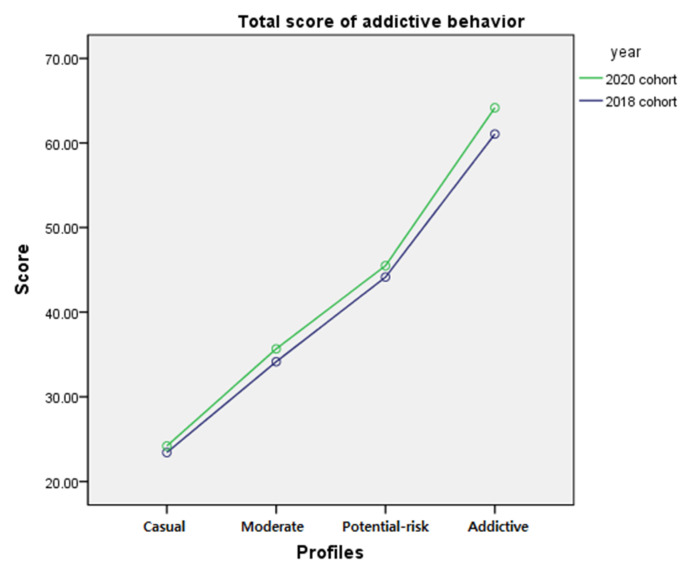
Interaction of profile membership and year on total score of addictive internet game usage.

After the optimal number of profiles were selected for the 2018 and 2020 cohorts, differences in total addictive internet gaming behavior, time spent playing internet games the on a PC, mobile gaming, and total time spent playing games on the internet, by year and profile membership were examined. First, differences in total addictive internet gaming behavior by profile membership before and after the COVID-19 pandemic were examined by a two-way ANCOVA after controlling for gender and school level. The results showed a significant interaction (*F* = 9.518, *p* < 0.001) between year and profile membership and significant main effect of year (*F* = 119.747, *p* < 0.001) and profile membership (*F* = 15316, *p* < 0.001). The interaction effect showed that the difference in gameplay time between the 2018 and 2020 cohorts was greater for profiles with higher addictive behavior scores. The main effect indicated that the total scores in 2020 were significantly greater than the total scores in 2018. To examine the differences among the four profiles, a post hoc test was performed. Results indicated significant differences among the profiles, with the ‘addictive’ gamer spending the highest time playing internet games, followed by ‘potential-risk’ gamer, ‘moderate’ gamer and ‘casual’ gamer (*p* < 0.05). Results from the post hoc test implied that the total score of addictive internet game usage significantly differs among the profiles. However, the mean difference of profiles between the 2018 and 2020 cohorts ranged from 0.76 to 3.11, which is the same as the 0.04 to 0.15 score difference on a four-point Likert scale. The results are presented in Table 4. The mean and standard error of internet gaming time by year and profiles are presented in Table 5 and the interaction effect is presented in Figure 3.

**Figure 4:**
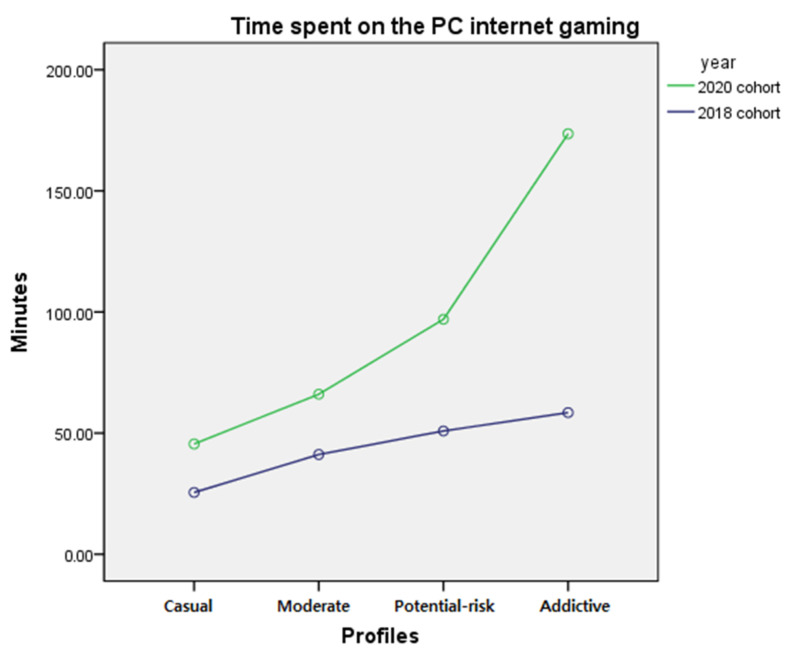
Interaction of profile membership and year on PC internet gaming time.

The difference in the time spent playing internet games on a PC by profile membership before and after the COVID-19 pandemic was examined by two-way ANCOVA after controlling for gender and school level. The results showed a significant interaction (*F* = 22.893, *p* < 0.001) between year and profile membership and a significant main effect of year (*F* = 198.21, *p* < 0.001) on the profile membership (*F* = 99.484, *p* < 0.001). The interaction effect showed that the difference in gameplay time between the 2018 and 2020 cohorts was greater for profiles with higher addictive internet game usage scores. To examine the differences among the four profiles, a post hoc test was performed; results indicated significant differences among profiles with the ‘addictive’ gamers who spent most time playing games, followed by the ‘potential-risk’ gamers, ‘moderate’ gamers, and ‘casual’ gamers (*p* < 0.05). The result of the post hoc test implies that those belonging to profiles with a higher addictive internet gaming usage spend more time playing internet games on a PC, regardless of the year. The results are presented in Table 6. The mean and standard error of the internet gaming time by year and profiles are presented in Table 7 and the interaction effect is presented in Figure 4.

**Figure 5:**
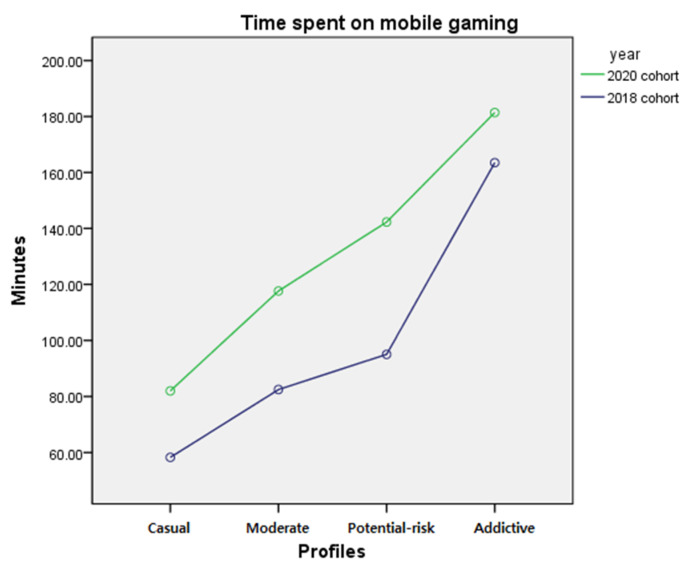
Interaction of profile membership and year on mobile gaming time.

The difference in the time spent on mobile gaming by profile membership before and after the COVID-19 pandemic was examined by two-way ANCOVA after controlling for gender and school level. The results showed a significant interaction between year and profile membership (*F* = 3.245, *p* < 0.05) and a significant main effect of year (*F* = 34.527, *p* < 0.001) on profile membership (*F* = 79.557, *p* < 0.001). The interaction effect demonstrated that the difference of gameplay time between the 2018 and 2020 cohorts was greater for profiles with higher addictive internet game usage, except for ‘addictive’ gamers. To examine the differences among the four profiles, a post-hoc test was performed. Results indicated significant differences among the profiles, with ‘addictive’ gamers spending the most time playing games, followed by the ‘potential-risk’ gamers, ‘moderate’ gamers and ‘casual’ gamers (*p* < 0.05). The post hoc test results implied that the individuals profiled with higher addictive internet gaming usage spent more time on mobile gaming, regardless of the year. The results are presented in Table 8. The mean and standard error of the internet gaming time by year and profiles are presented in Table 9 and the interaction effect is presented in Figure 5.

**Figure 6:**
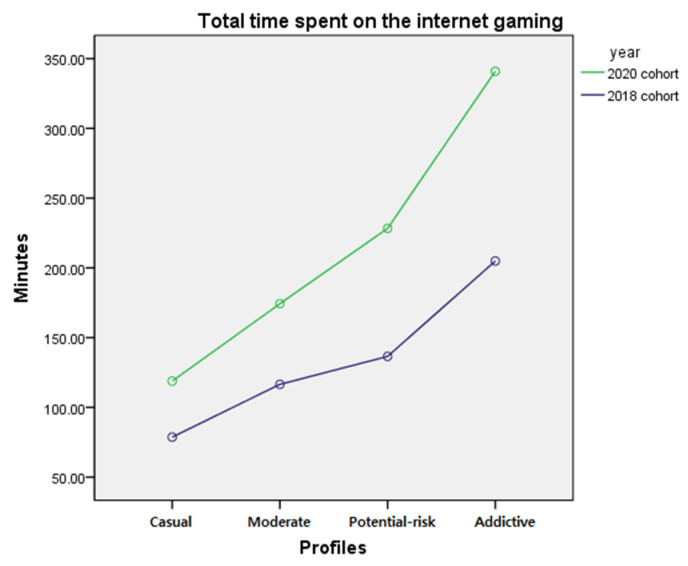
Interaction of profile membership and year on total time spent on gaming.

The difference in total time spent on internet gaming by profile membership before and after the COVID-19 pandemic was examined by two-way ANCOVA after controlling for gender and school level. The results showed a significant interaction (*F* = 44.059, *p* < 0.001) between year and profile membership and a significant main effect of year (*F* = 172.785, *p* < 0.001) and profile membership (*F* = 166.812, *p* < 0.001). The interaction effect showed that the difference in gameplay time between the 2018 and 2020 cohorts was greater for profiles with higher addictive internet game usage. To examine the difference among the four profiles, a post hoc test was performed. Results indicated significant differences among the profiles, with ‘addictive’ gamers spending the most time playing games, followed by ‘potential-risk’ gamers, ‘moderate’ gamers and ‘casual’ gamers (*p* < 0.05). The post hoc test results imply that individuals belonging to profiles with a higher addictive internet gaming usage spent more time on internet gaming, regardless of the year. The results are presented in Table 10. The mean and standard error of time spent on internet gaming by year and profiles are presented in Table 11, and the interaction effect is presented in Figure 6.

## 4. Discussion

There is increased concern about developing behavioral addictions due to the impacts of the COVID-19 pandemic, and adolescents may be more vulnerable to IGD [12,15]. This is especially true for adolescents in South Korea, which has one of the highest internet access rates and smartphone penetration rates across the world [39]. However, despite the impact of the pandemic on the playing of internet games and studies exploring internet game addiction [17,18,56], to the best of our knowledge, no study has been conducted on this topic in South Korea. Thus, the aim of this study was to estimate the profile of addictive internet gaming usage among South Korean adolescents in the 2018 and 2020 cohorts and explore how the severity of usage and gameplay time differed before and after the COVID-19 pandemic.

The results of LPA produced four profiles: ‘casual’ gamer, ‘moderate’ gamer, ‘potential-risk’ gamer, and ‘addictive’ gamer. The proportion of profiles indicates that more than 76% of the adolescents are playing internet games in a safe manner and approximately 21% show more involved gameplay, while only 3.5% of the 2018 cohort and 2.3% of the 2020 cohort exhibited a possible risk of IGD.

Despite the small proportion of the ‘addictive’ gamer profile, it can be highly problematic. As presented in Figure 4 and Figure 6, gameplay time increased significantly after the COVID-19 pandemic with an overall increase of 136.02 min, increasing the average play time approximately 6 h per day on weekdays. Since March 2020, schools were closed, and online teaching has been implemented since. All adolescents had to use a PC to take online classes and this might have increased the opportunity for adolescents to play internet games instead of focusing on classwork. As Triberti et al. [45] suggests, morning time, which is typically reserved for school for adolescents, may become gameplay time for highly addicted gamers. It may also explain the high increase of gameplay time in high-addiction profiles as they cannot resist the urge to play games during online schooling, while most nonaddictive adolescents will perform regular activities such as online schooling [45].

Moreover, the ‘addictive’ gamer profile showed the longest play time on both a PC (173 min) and mobile phones (183 min). Gamers with consistently high gaming time using both a PC and smartphone are reported to exhibit a higher prevalence of IGD in South Korea [52]. Also, they may be at a greater risk of developing depression, an anxiety disorder, or a substance disorder [55], which was reported to be more prevalent during the COVID-19 pandemic [1,18]. Applying the I-PACE model [35], the ‘addictive’ gamer profile may be in a ‘later stage’ of behavior addiction with the presence of strong addiction symptoms of habituation and extremely long gameplay time under stress-inducing environments during the COVID-19 pandemic, which may possibly require clinical intervention. Moreover, if gaming behavior becomes habitual, it may be more difficult for adolescents with the ‘addictive’ gamer profile to adapt to the environment when the pandemic is over. However, it will be crucial for policy makers, especially in education, to place emphasis on the approximately 97% of the nonaddictive adolescent gamers. As the results of the present study suggest, most adolescents play games on the internet regardless of gender, and the proportion of adolescent internet gamers is increasing rapidly. This implies that educational measures rather than preventative measures for adolescent gamers will be more beneficial. However, a study about teachers’ perception and acceptance of students’ gaming activities indicated that about 61.6 % of teachers had negative perceptions regarding gameplay [57]. This implies that educational measures regarding game usage need to be implemented for both students and teachers in South Korea.

The proportion of the IGD group in this study is similar to the global prevalence of 1.96% [8] and relatively smaller than the prevalence reported in a German study (5.6%) [58], but much lower than the 17% [42] IGD prevalence reported in China. The proportion of the IGD risk group was similar to that of other studies using the LPA score of IGD measuring scales. The proportion of the IGD group in one global study, with participants from 57 countries, was 5.3% and a study conducted in the Netherlands demonstrated a prevalence rate of 4.9% [8,48]. Despite high internet accessibility, high smartphone penetration rates, and raised concerns of IGD [30] in South Korea, the proportion of the IGD risk group in this study was close to the global prevalence [10] and lower than that of China. The gender difference in prevalence of IGD was similar to that of previous studies [9,10], which found male adolescents to be more likely to belong to a profile with higher addictive behavior. However, caution should be exercised when comparing the proportion of this study directly to other studies due to a large variability among studies [9,10].

Comparing the proportion of the profiles from the 2018 and 2020 cohorts, the portion of the ‘casual’ gamer is lower in the 2020 cohort by 3%, while the proportion of the ‘moderate’ gamer is greater in the 2020 cohort by 0.7% and the ‘potential-risk’ gamer is greater in the 2020 cohort by 4.3%. The proportion of the ‘addictive’ gamer is smaller in the 2020 cohort by 1.2%. These differences may indicate that the proportion of groups with a higher risk of IGD decreased, while the proportion of other profiles increased in the 2020 cohort. However, these differences in proportions are not obtained from statistical inference. Additionally, the present study compared the two different cohorts from two different time periods but did not compare the same individuals. Thus, such a relatively small difference in proportions might not have been caused by the impact of the COVID-19 pandemic and should be interpreted with caution.

The total score of addictive internet gaming usage between the 2018 and 2020 cohorts showed a statistically significant difference, but the mean difference among the profiles were 0.76 in ‘casual’, 1.51 in ‘moderate’, 1.34 in ‘potential-risk’, and 3.11 in ‘addictive’ profiles. On a four-point Likert scale, such differences indicate differences of 0.04, 0.07, 0.06 and 0.15 points, respectively. The results indicate that the majority of adolescents, except for the ‘addictive’ gamers, did not exhibit significant aggravation of addictive internet gaming usage during the COVID-19 pandemic. Approximately 2.3% of those fitting into the ‘addictive’ gamer profile may need immediate intervention of mental health professionals, but access to therapy or counseling services has been limited due to governmental regulations such as quarantine and social distancing [59,60]. Although there are governmental counseling services in which counselors visit adolescent clients [61], there are still insufficient counselors available, and most importantly, the danger of COVID-19 infection still exists. In such cases, telephone or online counseling can be used and parents of adolescents can utilize behavior intervention by following recommendations in lifestyle and internet usage [22]. For parents, monitoring strategies may be effective in preventing IGD during COVID-19 [62].

PC internet game time and mobile phone game time showed different trends in the 2018 and 2020 cohorts. The differences showed an increasing trend in accordance with the order of severity of each profile’s addictive behavior in time spent on a PC internet game. Mobile phone game time was different between the 2018 and 2020 cohorts and the degree of change differed according to the profiles. Gameplay time on mobile phones was higher in the 2020 cohort compared with the 2018 cohort in all profiles; the differences were higher in the order of ’potential-risk’, ‘moderate’, and ‘casual’ profiles, and the lowest difference was found in the ‘addictive’ profile. Although the cause of such a trend difference is not explored in this study, one possible explanation is that games played on a PC versus mobile phones may fulfill different demands [52].

The increasing trend of gameplay time on mobile phones is evident, and the reason for the increased play time may be partly due to the impact of the COVID-19 pandemic. The mobile gaming industry has been growing drastically since its introduction with smartphones and earned a total revenue of $86.3 billion in 2020 [63]. As the gaming industry continues to prosper during the COVID-19 pandemic, it can be expected that, unlike internet gaming on a PC, the mobile phone gaming industry will continue to grow, attracting more adolescent players. Because of the easy accessibility of mobile phone games, play time may not decrease after the pandemic is over. Despite its popularity, mobile phone gaming has not received much attention in research compared with other forms of gaming, such as PC and game consoles in South Korea [64,65,66,67]. Considering it is a rapidly growing industry with a high ratio of mobile phone gamers among Korean adolescents, more extensive research is needed.

Although the results of the present study indicate that profiles with higher addictive internet gaming usage exhibit longer gameplay time, caution should be exercised when interpreting higher gameplay time as problematic. There are cases where high involvement in gaming is not the same as problematic gaming [43,44,45,46,66]. Further, the IGD scale development study revealed that two distinct profiles—one with low risk and high engagement and one with high risk and high engagement—exist among adolescent gamers [48]. Most significantly, playing games online should not be stigmatized as gaming is not pathologic and it does have positive effects [23,24,25,26]. In addition, games can be utilized in educational purposes such as a ‘serious game’ [68].

Finally, there are limitations of the present study. First, a longitudinal comparison was not possible as the cohorts of 2018 and 2020 were different. As a direct comparison was not possible, the results should be interpreted as the difference between two different cohorts during two different time periods. Thus, the results comparing the 2018 and 2020 cohorts of the present study should not be interpreted as an increase or decrease, but should be interpreted as the difference between two different time points. If the survey was conducted longitudinally, a more meaningful conclusion might have been drawn. Second, the scale used in this study was not a scale that explicitly measures IGD symptoms but measures GD symptoms in general, which influences and limits the interpretation of the present study. Third, this study did not include internet gaming through consoles. However, considering the gaming population in South Korea, console games might not have had significant influence on adolescent gamers in South Korea. According to a report by the Korea Creative Content Agency in 2020, 10.4% of adolescents, which is the lowest ratio compared to other age groups, except those in their 50s or older, confirmed that they play console games [37]. The proportion may be smaller in the case of playing internet games using a console, which is rare in South Korea.

## Figures and Tables

**Table 1:** Correlation between seven MGUS factors and total gaming time.

	Tolerance 1	Withdrawal 2	Excessive Usage 3	Control Impairment 4	Compulsive Usage 5	Neglecting Activity 6	Negative Consequence 7
1	1						
2	0.721 ***	1					
3	0.665 ***	0.584 ***	1				
4	0.622 ***	0.612 ***	0.718 ***	1			
5	0.675 ***	0.667 ***	0.619 ***	0.641 ***	1		
6	0.617 ***	0.651 ***	0.493 ***	0.551 ***	0.613 ***	1	
7	0.669 ***	0.669 ***	0.642 ***	0.649 ***	0.667 ***	0.688 ***	1
Total gaming time	0.271 ***	0.225 ***	0.266 ***	0.244 ***	0.358 ***	0.266 ***	0.278 ***

*** *p* < 0.001.

**Table 2:** Fit indexes for the 2018 and 2020 cohorts.

Profile #	AIC	BIC	SABIC	Entropy	LMR-LRT, *p*-Value	BLRT, *p*-Value
Year 2018 Cohort						
2	73076.62	73209.05	73139.14	0.949	0	0
3	69917.63	70098.22	70002.90	0.952	0.001	0
4	68186.72	68415.46	68294.72	0.920	0.009	0
5	67540.25	67817.15	67670.99	0.922	0.352	0
6	66912.49	67237.85	67066.27	0.924	0.555	0
**Year 2020 Cohort**						
2	72466.88	72598.31	72528.41	0.928	0	0
3	69894.28	70073.52	69978.20	0.935	0.008	0
4	68663.18	68890.21	68769.47	0.893	0.052	0
5	68128.04	68402.87	68256.71	0.894	0.599	0
6	67476.44	67799.06	67627.48	0.901	0.003	0

Notes: AIC: Akaike information criterion; BIC: Bayesian information criterion; SABIC sample size adjusted BIC; LMR-LRT: Lo-Mendell-Rubin adjusted likelihood ratio test; BLRT: bootstrap likelihood ratio test.

**Table 3:** Addictive internet gaming usage and standard error of each profile of the 2018 and 2020 cohorts.

	‘Causal’ Gamer	‘Moderate’ Gamer	‘Potential-Risk’ Gamer	‘Addictive’ Gamer
Year 2018 Cohort				
Tolerance	3.38 (0.02)	4.87 (0.07)	6.34 (0.08)	9.17 (0.32)
Withdrawal	3.16 (0.01)	4.12 (0.07)	5.89 (0.08)	8.42 (0.30)
Excessive usage	3.61 (0.03)	6.38 (0.08)	7.17 (0.10)	9.52 (0.29)
Control impairment	3.34 (0.02)	5.43 (0.08)	6.54 (0.10)	9.12 (0.34)
Compulsive usage	3.44 (0.03)	4.93 (0.06)	6.48 (0.08)	9.20 (0.22)
Neglecting daily activity	3.54 (0.01)	3.70 (0.04)	5.32 (0.04)	7.14 (0.33)
Gaming despite negative consequence	3.33 (0.02)	4.65 (0.05)	6.42 (0.08)	8.67 (0.25)
Total	23.43(0.09)	34.15(0.14)	44.14(0.14)	61.06(0.34)
**Year 2020 Cohort**				
Tolerance	3.47 (0.03)	5.06 (0.09)	6.44 (0.11)	9.19 (0.46)
Withdrawal	3.21 (0.02)	4.25 (0.11)	6.10 (0.12)	8.81 (0.38)
Excessive usage	3.86 (0.06)	6.63 (0.09)	7.40 (0.11)	9.73 (0.48)
Control impairment	3.43 (0.04)	5.77 (0.11)	6.92 (0.13)	9.57 (0.34)
Compulsive usage	3.59 (0.04)	5.20 (0.10)	6.64 (0.11)	9.44 (0.35)
Neglecting daily activity	3.19 (0.02)	3.73 (0.12)	5.26 (0.05)	7.98 (0.52)
Gaming despite negative consequence	3.43 (0.03)	5.00 (0.12)	6.64 (0.11)	9.46 (0.32)
Total	24.19(0.09)	35.66(0.13)	45.48(0.14)	64.17(0.42)

**Table 4:** Two-way ANCOVA: total score of addictive internet gaming usage by year and profile membership.

	SS	df	MS	F	η^2^
Membership	552434.107	3	184144.702	15316.560 ***	0.886
year	1439.667	1	1439.667	119.747 ***	0.020
Membership × year	330.300	3	110.100	9.158 ***	0.005

*** *p* < 0.001.

**Table 5:** Mean and standard error of total score of addictive internet gaming usage by profile membership and year.

	‘Casual’	‘Moderate’	‘Potential-Risk’	‘Addictive’	Mean
2018 cohort	23.43(0.09)	34.15(0.14)	44.14(0.14)	61.06(0.34)	40.69(0.10)
2020 cohort	24.19(0.09)	35.66(0.13)	45.48(0.14)	64.17(0.42)	42.78(0.12)
Mean difference	0.76	1.51	1.34	3.11	2.09

**Table 6:** Two-way ANCOVA: time spent playing internet games on a PC by year and profile membership.

	SS	df	MS	F	η^2^
Membership	1769595.249	3	589865.083	99.484 ***	0.053
year	1175243.349	1	1175243.349	198.210 ***	0.036
Membership × year	407217.691	3	135739.230	22.893 ***	0.013

*** *p* < 0.001.

**Table 7:** Mean and standard error of time spent on playing internet games on a PC by profile membership and year.

	‘Casual’	‘Moderate’	‘Potential-Risk’	‘Addictive’	Mean
2018 cohort	25.51(2.01)	41.16(3.17)	50.89(3.34)	58.46(8.38)	44.01(2.44)
2020 cohort	45.52(2.15)	66.06(2.94)	96.98(3.26)	173.56(9.79)	95.53(2.74)
Mean difference	20.01	24.9	46.09	115.10	51.52

**Table 8:** Two-way ANCOVA: time spent playing internet games on a PC by year and profile membership.

	SS	df	MS	F	η^2^
Membership	3140362.077	3	1046787.359	79.557 ***	0.041
year	454302.916	1	454302.916	34.527 ***	0.006
Membership × year	128106.403	3	42702.134	3.245 *	0.002

*** *p* < 0.001, * *p* < 0.05.

**Table 9:** Mean and standard error of time spent on a PC internet gaming by membership and year.

	‘Casual’	‘Moderate’	‘Potential-Risk’	‘Addictive’	Mean
2018 cohort	58.27	82.48	95.03	163.50	99.82
2020 cohort	81.99	117.62	142.28	181.43	130.83
Mean difference	23.72	35.14	47.25	17.93	31.01

**Table 10:** Two-way ANCOVA: total time spent on the internet gaming by year and profile membership.

	SS	df	MS	F	η^2^
Membership	9445189.083	3	3148396.361	166.812 ***	0.079
year	3261123.442	1	3261123.442	172.785 ***	0.029
Membership × year	831571.427	3	277190.476	44.059 ***	0.008

*** *p* < 0.001.

**Table 11:** Mean and standard error of total time spent on internet gaming by membership and year.

	‘Casual’	‘Moderate’	‘Potential-Risk’	‘Addictive’	Mean
2018 cohort	78.67(3.42)	116.52(5.39)	136.55(5.53)	204.94(13.39)	134.17(3.96)
2020 cohort	118.83(3.68)	174.34(5.03)	228.30(5.63)	340.96(17.18)	215.61(4.78)
Mean difference	40.16	57.82	91.75	136.02	81.44

## Data Availability

Not applicable.
